# Noise Reduction in Brainwaves by Using Both EEG Signals and Frontal Viewing Camera Images

**DOI:** 10.3390/s130506272

**Published:** 2013-05-13

**Authors:** Jae Won Bang, Jong-Suk Choi, Kang Ryoung Park

**Affiliations:** Division of Electronics and Electrical Engineering, Dongguk University-Seoul, 26 Pil-dong 3-ga, Jung-gu, Seoul 100-715, Korea; E-Mails: bangjw@dgu.edu (J.W.B.); jjongssuk@dgu.edu (J.-S.C.)

**Keywords:** EEG, BCI, LDA, SVM

## Abstract

Electroencephalogram (EEG)-based brain-computer interfaces (BCIs) have been used in various applications, including human–computer interfaces, diagnosis of brain diseases, and measurement of cognitive status. However, EEG signals can be contaminated with noise caused by user's head movements. Therefore, we propose a new method that combines an EEG acquisition device and a frontal viewing camera to isolate and exclude the sections of EEG data containing these noises. This method is novel in the following three ways. First, we compare the accuracies of detecting head movements based on the features of EEG signals in the frequency and time domains and on the motion features of images captured by the frontal viewing camera. Second, the features of EEG signals in the frequency domain and the motion features captured by the frontal viewing camera are selected as optimal ones. The dimension reduction of the features and feature selection are performed using linear discriminant analysis. Third, the combined features are used as inputs to support vector machine (SVM), which improves the accuracy in detecting head movements. The experimental results show that the proposed method can detect head movements with an average error rate of approximately 3.22%, which is smaller than that of other methods.

## 1. Introduction

An electroencephalogram (EEG) is a test during which the electric signals of the brain are measured from the scalp of the user. An EEG-based brain–computer interface (BCI) has been widely researched for various applications, including intuitive control for computers, mobile devices, wheelchairs, and robots, as well as for the diagnosis of cerebropathy and for the indirect measurement of the cognitive, emotional, and psychological status of the user [1–6]. In a previous research study, navigation in 3D applications was performed by combining gaze tracking and an EEG measuring device [4]. A research study showed that the EEG-based driver assistant system could perform emergency braking more quickly than that based on pedal responses [7]. Another study showed that the user's EEG signals can be used to control electric wheelchairs [2,8]. A study showed the results of controlling a BCI speller system based on the steady-state visually evoked potential (SSVEP) [9].

In previous studies, EEG measurement methods using a wearable device were classified into two categories: headset-based and electrode-cap-based methods [3]. The former uses a headset-type device mounted on the user's head [4,5,8,10], and the latter uses a swimming-cap-type EEG acquisition device [7,9,11]. In general, the electrode-cap-type device acquires higher quality EEG signals that are less affected by head movements, because it is tightly worn on the head and it has more electrodes than the headset-type device. However, the headset-type device has the advantages of lower cost and enhanced user convenience, because it can be worn easily. Therefore, in this study, we used the headset-type acquisition method for EEG signals.

In most cases using the headset-type method, the EEG signals can be easily contaminated with noise due to head movements or eye blinks [12], and this noise should be compensated for in order to acquire more reliable EEG signals. Head movements (rotation and translation of head) usually result in positional changes in a number of EEG electrodes on the head, thereby introducing artifacts into the EEG signals.

Research studies on detecting the EEG artifacts caused by head movements were previously conducted. In those studies, only the EEG signals were analyzed for estimating the noises induced by head movements or eye-blink [12–14]. The head movements were detected by using the extracted temporal-, frequency-, and entropy-based features of the EEG signals [13]. In [14], authors proposed the automatic classification method of general artifacts in EEG signals by using Temporal Decorrelation source SEParation (TDSEP), Independent Component Analysis (ICA), and Linear Programming Machine (LPM). However, they used only the features from EEG signals, and the performance enhancement can be limited compared to our method which uses both the features of EEG signals and frontal viewing camera images.

The head movements was estimated by analyzing the image sequences obtained by a frontal viewing camera, which was attached to a glass-type lightweight device [10], to enhance the detection accuracy. In another research, the artifacts caused by walking and running were studied [11]; however, high-density EEG signals were measured using 248 electrodes, which increased the device cost and rendered it highly inconvenient for the user [11]. O'Regan *et al.* proposed a new method to detect EEG artifacts caused by head movements by combining the features of the EEG and gyroscope signals [15]. Although the accuracy of that method is very high, it takes more time to extract 16 features (in the time and frequency domains) from the gyroscope signals than to extract only four features from the camera images used in our research (see Section 2.2.1). Sweeney *et al.* proposed a new method of removing artifacts in the EEG signals using a functional near-infrared spectroscopy (FNIRS) and an accelerometer [16]. However, it has the disadvantage that the setup of two accelerometers requires much care (to ensure the orientation of the two accelerometers is kept consistent with respect to each other [16]).

Our frontal viewing camera-based method is more flexible than the gyroscope-based and the accelerometer-based methods, because it can distinguish other types of head movements, including shaking, nodding, rolling, moving in horizontal and vertical directions, and moving closer to or farther from the monitor. By combining the features of the scene which the user is looking at (environmental information) with the EEG features, the accuracy of BCI in the real world can be enhanced.

The aim of this research is to detect and exclude the sections of EEG data containing the noises caused by head movements (*i.e.*, the head rotation (yaw) in the horizontal direction) by combining an EEG acquisition device and a frontal viewing camera. By excluding EEG signals that contain such artifacts, we can enhance the accuracy of the EEG-based computer interface system. Based on our experiments with a P300-based speller system, we confirmed the usefulness of the proposed method in a BCI system. Table 1 summarizes the comparison of previous methods and the proposed method.

The rest of this paper is organized as follows: in Section 2, the proposed system and method are described. In Section 3, the experimental setup and results are discussed. Finally, in Section 4, the conclusions are presented.

## 2. Proposed System and Method

### 2.1. Proposed Device and Speller UI System

**Figure 1.**
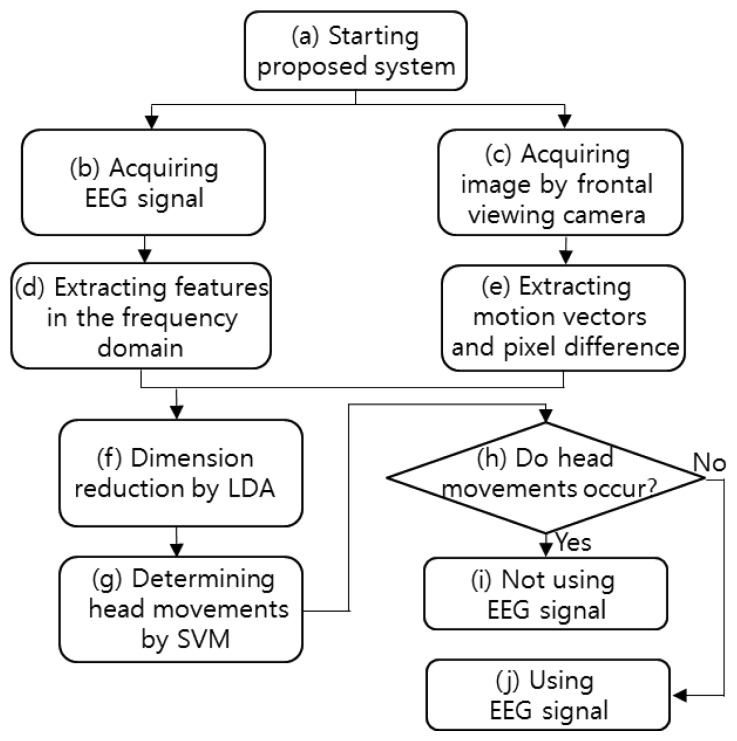
Flowchart of proposed system.

The flowchart of the proposed system is shown in Figure 1. After starting the proposed system, both the EEG signals and the image captured by the frontal viewing camera are acquired simultaneously. The features of the acquired EEG signals are extracted in the frequency domain, and the image features (pixel difference, edge pixel difference, average magnitude of the motion vectors, number of motion vectors) are extracted from the successive images captured by the frontal viewing camera. The EEG and image features are combined, and their dimensions are reduced by using linear discriminant analysis (LDA). The head movements are determined by the support vector machine (SVM) on the basis of the features of reduced dimensions. If head movement is found, the EEG signals are not used. On the contrary, if no head movement is found, the EEG signals are used.

**Figure 2.**
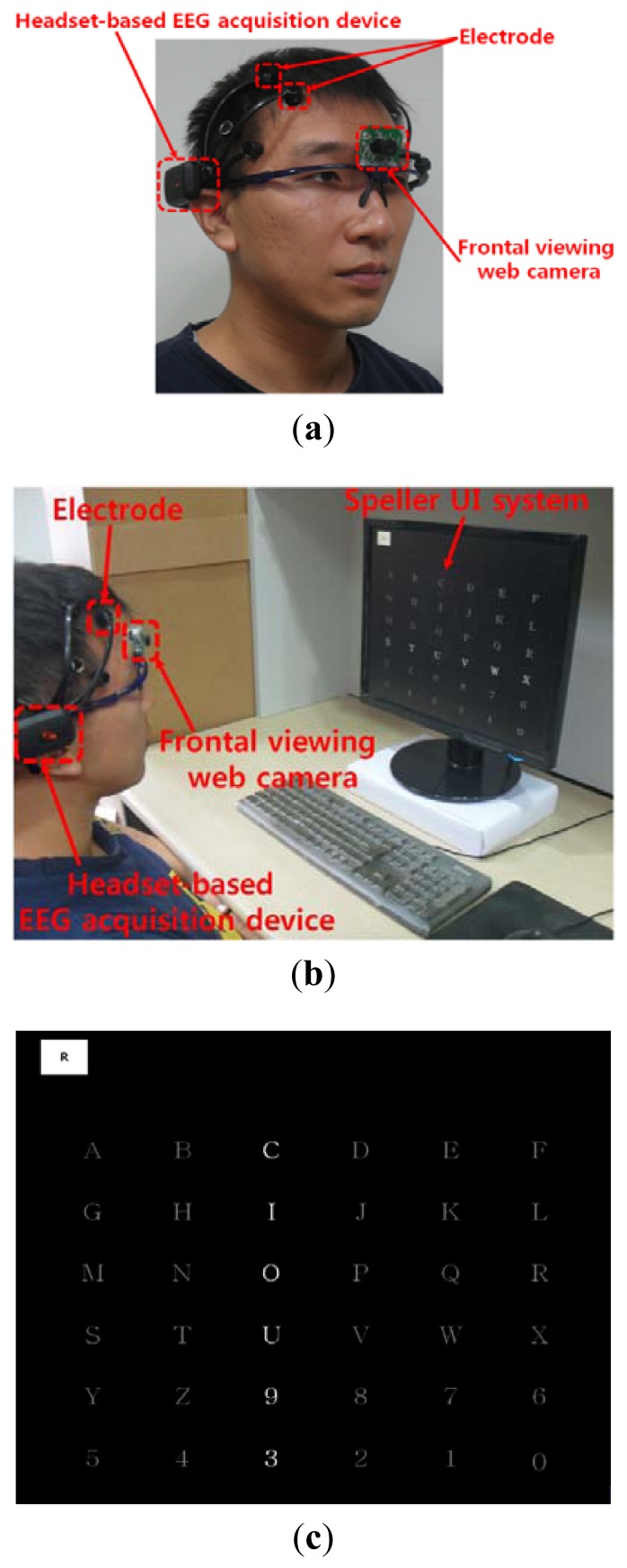
Proposed device and speller UI system. (**a**) Proposed device; (**b**) Example of experimental environment; (**c**) Speller UI system.

**Figure 3.**
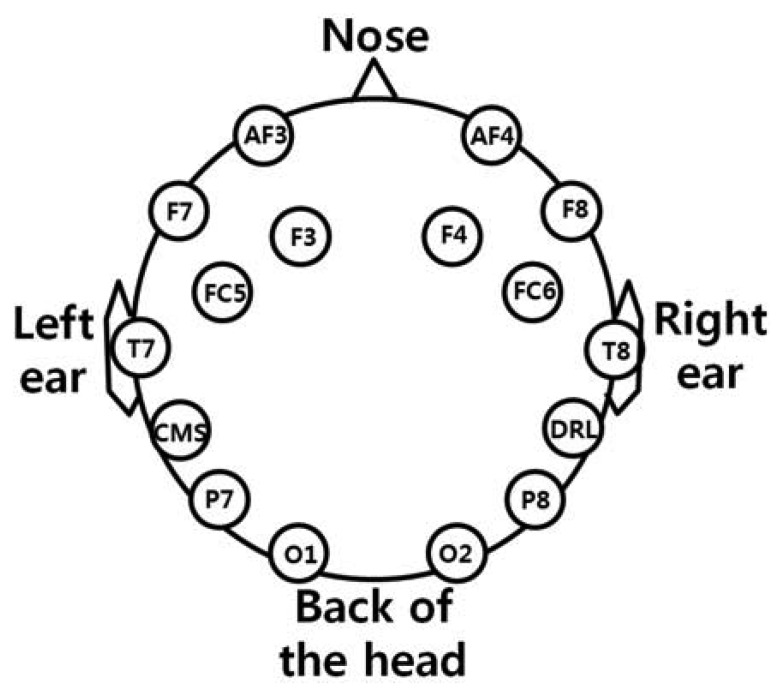
The positions of 16 electrodes of the Emotiv EPOC headset.

As shown in Figure 2(a), the user wears both the headset-type EEG measuring device and the frontal viewing camera mounted on an eyeglass-type frame [10]. A commercial Emotiv EPOC headset is used as the EEG measuring device, which is comprised of 16 electrodes with two reference nodes (CMS and DRL, as shown in Figure 3) [17]. The electrodes are positioned based on the international 10–20 system of electrode placement, also shown in Figure 3 [5,18]. The Emotiv EPOC system outputs the processed EEG data by using a built-in digital 5th order Sinc filter. Specific information about the impedance measurement was not disclosed [19]. In order to reduce the EEG noise of each electrode, we used the averaged value of the EEG data from the 14 electrodes. The sampling rate of the Emotiv EPOC system is 128 Hz (128 samples/s). The EEG data is shown as a floating-point number of 4 bytes, which represents the voltage of an electrode [5]. Because the frontal viewing camera acquires images at 30 Hz (30 frames/s), we used the EEG data which was obtained at the same moment when the image is acquired, in order to synchronize the EEG data and the camera images. As shown in Figure 2(b,c), we use the speller user interface (UI) system, which has been widely used for testing P300-based BCI [10,20]. A commercial web camera is used as the frontal viewing camera (Webcam C600) [21]. The captured image is 640 × 480 pixels with 24-bit RGB colors, acquired at 30 frames/s.

### 2.2. Feature Extraction

#### 2.2.1. Feature Extraction from EEG Signals

To decrease the variation in the EEG signals obtained from the user, we normalize the signals by adjusting the DC level (average value) of the EEG magnitude to 0, and then performing min-max scaling normalization to represent the EEG magnitude within the range from −1 to 1. As the features of the EEG signals, the normalized magnitudes in frequency domain are obtained from the corresponding frequency bands by using the Fourier transform (FT). We obtained the magnitudes at 12 frequency bands (3–15 Hz, 4–6 Hz, 5–7 Hz, 6–8 Hz, 7–9 Hz, 8–10 Hz, 9–11 Hz, 10–12 Hz, 15–30 Hz, 20–30 Hz, 51–64 Hz, 59–61 Hz). The frequency-domain features of Table 2 are obtained by applying FT on samples in a window with a length of 128 samples. This window is moved by overlapping 127 samples, and the frequency-domain features are consequently obtained every 128 samples.

The time-domain features of Table 2 are also obtained based on the window length of 128 samples. This window is moved by overlapping 127 samples, and the time-domain features are also obtained every 128 samples. Therefore, the epoch length in this research is 128 samples. The following thirteen features of the EEG signal are extracted in the time domain [13], and their performances are compared: kurtosis, skewness, root mean square (RMS) amplitude, zero-crossing, minimum, maximum, variance of first derivative, variance of second derivative, zero-crossing of first derivative, zero-crossing of second derivative, activity, mobility, and complexity. The features are calculated in the time domain after acquiring every 128 sample in the time domain.

Kurtosis shows the degree of the peak of a distribution, and it can represent the various kinds of distributions such as Laplace, logistic, normal, and uniform distributions, *etc.* [22]. Skewness shows the degree of the asymmetry of a distribution, where the left or right tail is relatively longer than the other [23]. The RMS amplitude is the peak value divided by $\sqrt{2}$ [24]. Zero-crossing point is that at which the sign of the EEG signal changes (from negative to positive or from positive to negative). We use the number of zero-crossing points during the predetermined period (128 samples of EEG data) as a feature.

Minimum and maximum are the minimum and maximum values, respectively, of the EEG signal in a particular interval. Variance of first derivative represents the varied range of the amplitude change (first derivative) of the EEG signal. Variance of second derivative shows the varied range of the first derivative change (second derivative) of the EEG signal.

Zero-crossing points of first derivative and second derivative are the changing points of sign (from negative to positive or from positive to negative) of the first and second derivative of the EEG signal value, respectively. We use the number of zero-crossing points during the predetermined period (128 samples of EEG data) as feature.

Activity represents the variance of the amplitude fluctuations in the EEG signal range. Mobility is the square root of the ratio of the variance of the first derivative of the EEG signal to the variance of the EEG signal. Complexity is the ratio of the mobility of the first derivative of the EEG signal to the mobility of the EEG signal [25,26]. All of these features in frequency- and time-domains are used, because they represent the global and local shapes of EEG data.

#### 2.2.2. Feature Extraction from Frontal Viewing Camera Image

As shown in Figure 2(a,b), the frontal viewing camera is mounted on an eyeglass-type frame, and the user's head movements cause the changes in the successive images captured by the camera. From the acquired frontal image sequences, we extracted four features, namely, pixel difference, edge pixel difference, average magnitude of the motion vectors, and number of motion vectors.

The pixel difference is the difference between the pixel values of successive images. Without any head movement, the pixel difference of successive images becomes smaller (similar to black pixel) because the pixel values of successive images at the same position are almost the same. With head movements, the pixel difference is greater.

To calculate the edge pixel difference, the Sobel operator is first applied to the images [27], and then the pixel difference between successive Sobel-processed edge images is calculated. We used two 3 × 3-pixel Sobel masks (horizontal and vertical directions). The edge pixel difference decreases if the user's head does not move, and the edge pixel difference is greater if the user's head moves.

The Lucas-Kanade-Tomasi (LKT) method is used to obtain the motion vectors from successive frontal images [10,28,29]:

(1) $$I_{t+1}(x)=I_{t}(x-d)+n(x)$$

In Equation (1), *I*_*t*+*1*_(***x***) and *I_t_*(***x***) are the brightness values in the next and current frames, respectively. ***x*** and ***d*** represent the vectors of pixel location and displacement, respectively. *n*(***x***) is the noise. The correlation of the two corresponding feature points in the two successive images is calculated based on the minimum mean squared error (MSE) (*E* of Equation (2)). In Equation (2), *W* is the feature window [29]:

(2) $$E=\sum_{i\in W}{\left(I_{t}(x_{i}-d)-I_{t+1}(x_{i})\right)}^{2}$$

The corresponding feature points and the motion vectors between these two feature points are obtained from the correlation result [10]. The magnitude and number of motion vectors decrease in the case of no head movements. With head movements, the magnitude and number of motion vectors are greater. The average magnitude of the motion vectors and the number of motion vectors are used as the third and fourth features of the frontal images, respectively. Table 2 lists all the features considered in this study.

### 2.3. Feature-Level Fusion and Determination of Head Movements

In this study, the multiple features listed in Table 2 are combined, and the optimal features of reduced dimensions are obtained by using LDA. LDA determines the optimal axes in terms of the classification by decreasing the within-class variance and increasing the between-class variance [30,31].

With the training samples, we can obtain the set of linear projection vectors ***u*** based on Fisher's criterion [32]:

(3) $$u=\arg\underset{u}{\max}\frac{\left|u^{T}M_{B}u\right|}{\left|u^{T}M_{U}u\right|}$$

**Figure 4.**
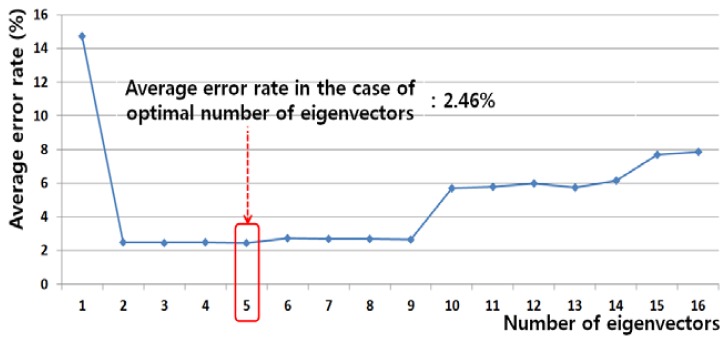
Example of using LDA to obtain the optimal number of feature dimensions from the training data, using both frontal image features and EEG features in the frequency domain.

In Equation (3), $M_{W}=\sum_{j=1}^{N}q_{j}M_{j}$ and $M_{B}=\sum_{j=1}^{N}q_{j}\left(m_{j}-m\right){\left(m_{j}-m\right)}^{T}$ are the matrices of the within-class scatter and between-class scatter, respectively. *N* is the number of classes. ***M****_j_* is the within-class scatter matrix of a specific class *j*. **m***_j_* and **m** are the mean vectors of data of the specific class *j* and all the training data, respectively. *q_j_* is the priori probability of data of the specific class *j* [32]. In this research, we used the unregularized LDA [33]. The optimal number of dimensions is experimentally determined in terms of the smallest average error as shown in Figure 4. The average error is the average value of Type 1 and Type 2 errors. Class 1 represents the case of no head movements and Class 2 represents the case of head movements. Type 1 error is the error of misclassifying Class 1 data as Class 2 data. Type 2 error is the error of misclassifying Class 2 data as Class 1 data.

The optimal features of reduced dimensions obtained by using LDA are used as the input to the SVM, and the final determination of the head movements is performed. The SVM, as a supervised learning model, is widely used for classifying two or more classes. The support vectors are used to detect the optimal decision hyperplane for the classification [34]. In general, SVM can be represented as follows [32,34]:

(4) $$f(x)=\mathrm{sgn}\left(\sum_{\forall x_{k}\in S}a_{k}y_{k}K(x_{k},x)+b\right)$$

where *S* denotes the set of support vectors, *α_k_* represents a training-derived weight, and *y_k_* = {−1, 1} denotes the class label of *x_k_*. We denote the Class 1 data as “−1” and the Class 2 data as “1” for SVM training. In this study, we used the LIBSVM program, and the optimal kernel parameter is experimentally determined from the training data, among the following kernels, as in Equation (5) [35]:

(5) $$\begin{array}{c} \text{Linear kernel}:K(x_{i},x_{j})=x_{i}^{T}x_{j}\\ \text{Polynominal kernel}:K(x_{i},x_{j})={\left(rx_{i}^{T}x_{j}+c\right)}^{d}\\ \text{RBF kernel}:K(x_{i},x_{j})=\exp(-r{\left\|x_{i}-x_{j}\right\|}^{2})\\ \text{Sigmoid kernel}:K(x_{i},x_{j})=\tanh(rx_{i}^{T}x_{j}+c) \end{array}$$

The final determination of the head movement is performed based on the SVM output value. The optimal threshold for the final determination is obtained in terms of the smallest average error of Type 1 and 2 errors.

## 3. Experimental Results

The experiments were performed on a desktop computer equipped with a 2.3 GHz CPU and 4 GB RAM. The proposed algorithm was implemented as C++ applications using the Microsoft Foundation Class (MFC) and OpenCV. Ten subjects participated in the experiments (average age: 26.5; standard deviation: 1.86). We acquired the EEG signals and frontal images when the subject's head did not move (Class 1) and when it moved (Class 2), respectively. Each of the subjects underwent the trial twice for the experiments. That is, each subject did two trials while he did not move his head, and he also did two trials while he naturally moved his head. In this research, the head movements included only the head rotation (yaw) in the horizontal direction.

The time period of one trial of either Class 1 or Class 2 was 45–50 s. During each period of Class 1 and Class 2, the average numbers of acquired EEG data are about 1,638 and 1,651, respectively, which are used for experiments. Before the trials, we gave the following instructions to each subject:

- For Class 1: “Please do not move your head at all.”
- For Class 2: “Please rotate your head in the horizontal direction naturally.”

With only these instructions, each subject performed two trials for Class 1 and two trials for Class 2. The time interval between each trial was approximately 1 min. The EEG data from the 1st and 2nd trials when the subject did not move his head were annotated as Class 1 data. The EEG data of the 1st and 2nd trials when he naturally rotated his head were annotated as Class 2 data.

**Figure 5.**
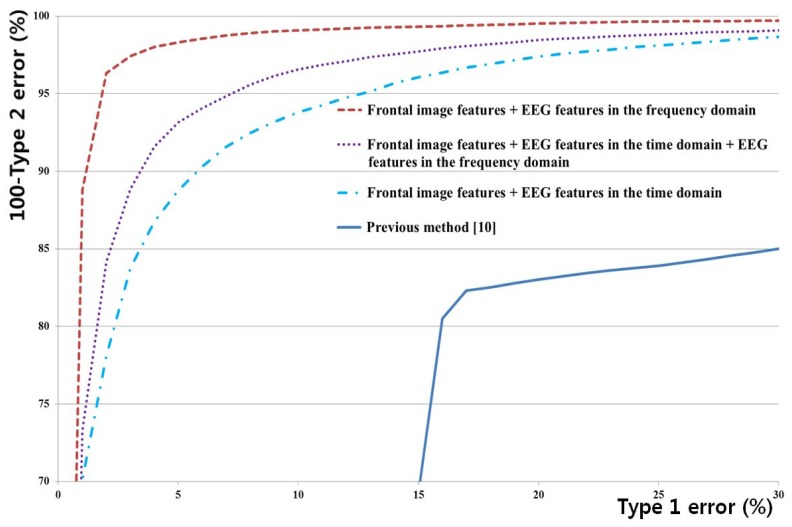
ROC curves for all methods.

We performed two-fold cross-validation to measure the accuracy of the methods. Half of the acquired data was used for LDA and SVM training, and the other part was used for testing in order to accurately measure the performance without the effects of the training data. Then, we switched the training data and the testing data with each other (*i.e.*, we used the original training data as the testing data and the original testing data as the training data) and performed the same procedures. We obtained the average accuracies of the two testing data sets as shown in Tables 3, 4, 5 and 6, and Figure 5. This scheme is named as cross-validation and has been widely used for pattern classification [36,37].

Because one trial data of the ten participants were used for training and the other trial data were used for testing, our classifier is participant-dependent. Each feature (Table 2) could have different values in each trial. However, these variations could be compensated for by using LDA and SVM to discriminate Class 1 and Class 2.

Table 3 summarizes Type 1 and Type 2 errors in the testing data using only LDA, without the SVM classifier. The optimal features of reduced dimensions were obtained from the training data of each of the combined features listed in Table 3, and the trained LDA classifier was applied to the testing data of each of the combined features. The Type 1, Type 2, and average errors were obtained, as summarized in Table 3. All the features are explained in Table 2. As summarized in Table 3, the average error rate is the lowest for the combination of the frontal image features and EEG features in the frequency domain. Figure 4 shows an example of using LDA to obtain the optimal number of feature dimensions from the training data in case of the first trial of two-fold cross-validation, based on the average error rate (in the case of using both the frontal image features and EEG features in the frequency domain). In addition, the optimal kernel with parameter and threshold of SVM are determined with training data.

Among the all the methods listed in Table 3, the last three ones (Frontal image features + EEG features in the time domain, Frontal image features + EEG features in the frequency domain, Frontal image features + EEG features in the time domain + EEG features in the frequency domain) showed smaller average errors compared to the first three methods (EEG features in the frequency domain, EEG features in the time domain, EEG features in the frequency domain + EEG features in the time domain). Therefore, with the last three methods, the final Type 1, Type 2, and average errors in the testing data were measured through LDA and SVM, as summarized in Table 4.

By comparing Table 3 and Table 4, we can confirm that the errors obtained by using both LDA and SVM together are smaller than those obtained by using only LDA. The kernel function with the parameter of the SVM in case of the lowest error rate (combining the frontal image features and the EEG features in the frequency domain, as shown in Table 4) is the RBF kernel with the parameter *r* value of 8.0 in Equation (5).

LDA has the characteristics of linear classifier. So, it cannot show the good classification performance with the data of complicated distributions whereas the non-linear classifier like SVM is usually reported to show the better performance in this case. In general, the transformed data by LDA (the projected data on LDA eigenvectors) shows the more separable characteristics based on each class than the original data. So, like PCA (principal component analysis), LDA can be used for selecting the optimal feature with dimension reduction before classification [31,38,39]. In previous researches, they also used this kind of scheme [36,39,40]. In [36], for age classification, they performed SVM-based classification by using the features obtained by PCA. In [39], for detecting epileptic seizure based on EEG signal, they also performed SVM-based classification by using the features obtained by LDA. In [40], they obtained the features by PCA+HMM (hidden markov model), and performed SVM-based classification for the discrimination of EEG signal acquired during the imagination of left or right hand movement. We did additional experiments to compare the performance of only using SVM to that using the proposed LDA+SVM. Comparing Tables 4 and 5, the average error of LDA+SVM is smaller than that only using SVM.

Figure 5 shows the receiver operating characteristic (ROC) curves of the testing data by using each of the abovementioned methods. As shown in Figure 5, we can confirm that the method that combines the frontal image features and the EEG features in the frequency domain achieves the highest accuracy compared to the other methods. In addition, the accuracy of the proposed method is higher than that of the previous method [10]. In another research study [13], various EEG signal-based features such as the time domain, frequency domain, and entropy-based features were used. In comparison with the average error in detecting the EEG artifacts in [13], the average error obtained with the proposed method (3.22%) is much smaller than that obtained with the previous method (∼23.5%).

Table 6 lists the Type 1, Type 2, and average errors when using both the frontal image features and EEG features in the frequency domain with LDA and SVM, for each subject. The average error value varies from person to person; however, we can confirm that the average error rate is low.

**Figure 6.**
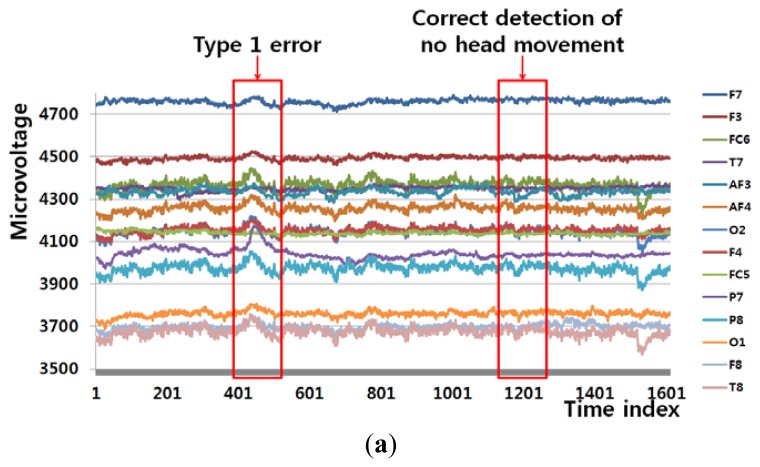

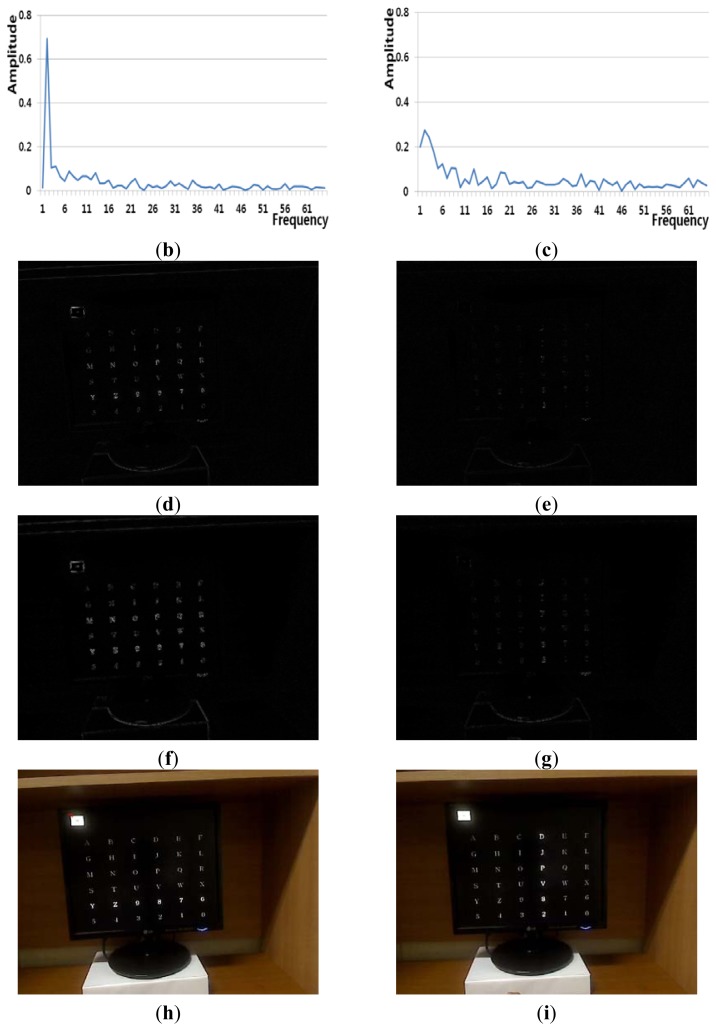
Example of a Type 1 error and the correct detection of no head movement (person 6 listed in Table 6). (**a**) EEG signals. (**b**), (**d**), (**f**), and (**h**) respectively show signals obtained from FT of EEG signals, pixel difference, edge pixel difference, and motion vectors using LKT in case of Type 1 error. (**c**), (**e**), (**g**), and (**i**) respectively show signals obtained from FT of EEG signals, pixel difference, edge pixel difference, and motion vectors using LKT in case of correct detection of no head movement.

Figure 6(a) shows an example of a Type 1 error and an example of the correct detection of no head movement (person 6 listed Table 6). In the case of no movement of the user's head (Class 1), the FT-processed EEG signal has a greater amplitude at low frequency and a lower amplitude at high frequency, as shown in Figure 6(b). However, the slight movements of the user's head can cause a small pixel and edge differences, as shown in Figure 6(d,f), and this, in turn, causes the Type 1 error.

Figure 6(c) also shows the case of no head movement (Class 1); however, the amplitude at low frequency is lower than that shown in Figure 6(b). These incorrect results in the frequency domain are caused by the variations in the EEG signals obtained from different users and by other factors, including the user's level of attention and environmental factors, such as lighting and noise. However, Figure 6(e,g,i) show a small amount of pixel, edge differences and motions, which causes the correct detection of no head movement.

**Figure 7.**
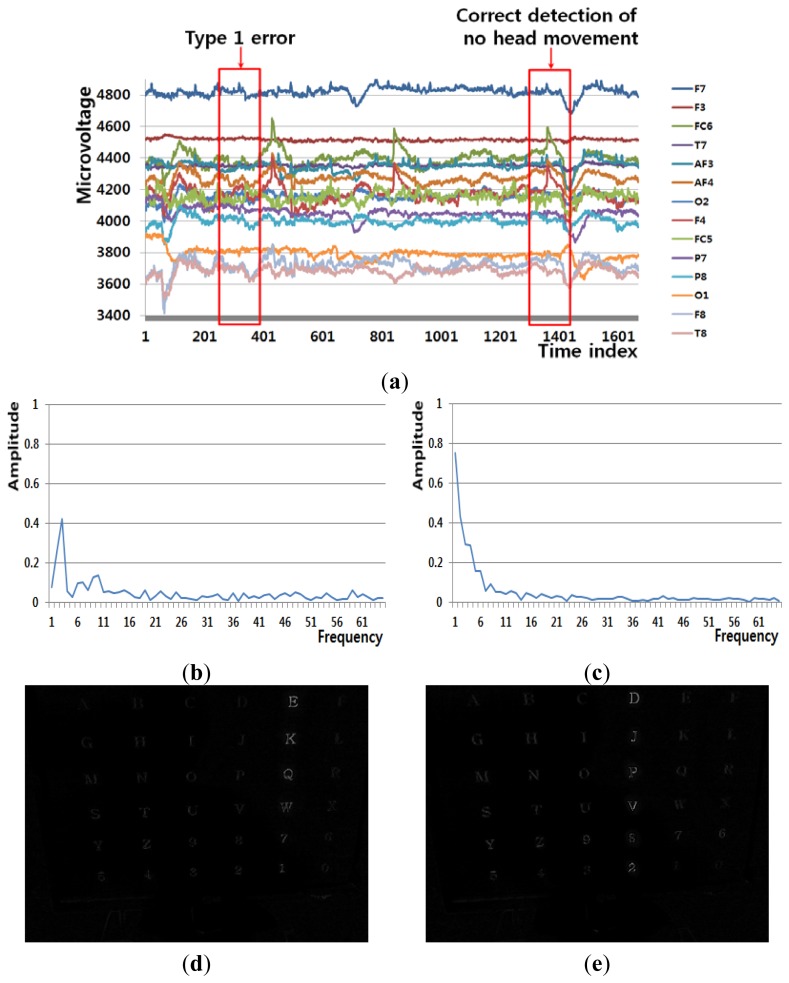

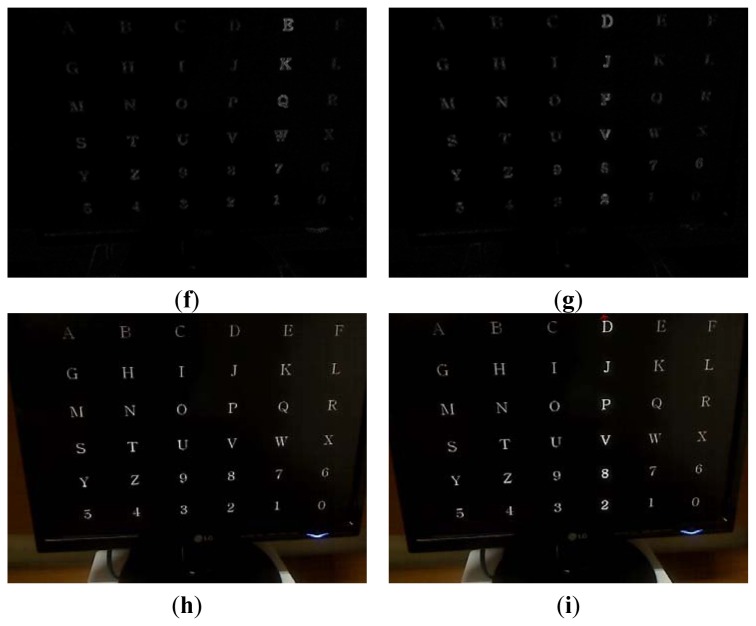
Example of a Type 1 error and the correct detection of no head movement (person 2 listed in Table 6). (**a**) EEG signals. (**b**), (**d**), (**f**), and (**h**) respectively show signals obtained from FT of EEG signals, pixel difference, edge pixel difference, and motion vectors using LKT in case of Type 1 error. (**c**), (**e**), (**g**), and (**i**) respectively show signals obtained from FT of EEG signals, pixel difference, edge pixel difference, and motion vectors using LKT in case of correct detection of no head movement.

Figure 7(a) shows an example of a Type 1 error and an example of the correct detection of no head movement (person 2 listed Table 6). Although it is the case of no head movement, the FT-processed EEG signal has a lower amplitude at low frequency as shown in Figure 7(b). In addition, the slight movements of the user's head cause the edge pixel difference as shown in Figure 7(f). These factors cause the Type 1 error of Figure 7(a). In case of the correct detection of no head movement of Figure 7(a), the FT-processed EEG signal correctly has a higher amplitude at low frequency as shown in Figure 7(c). In addition, the amount of pixel differences and motions of Figure 7(e,i) are smaller. These factors cause the correct detection of no head movement.

Consequently, correct detection of no head movements is achieved by combining the frontal image features and the EEG features in the frequency domain and then processing them using LDA and SVM.

**Figure 8.**
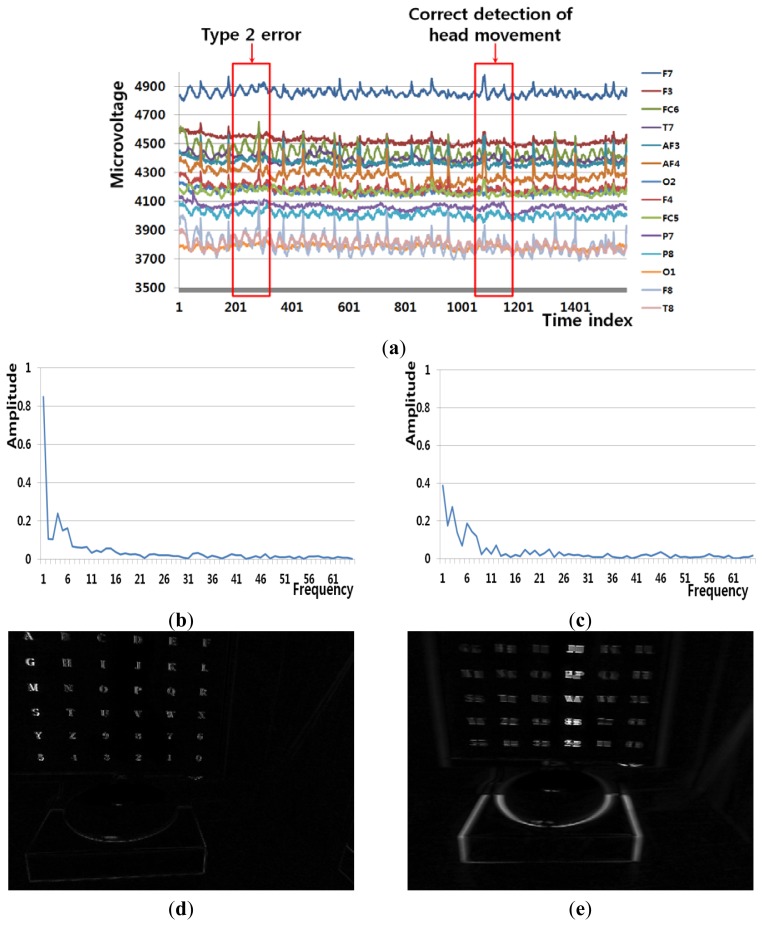

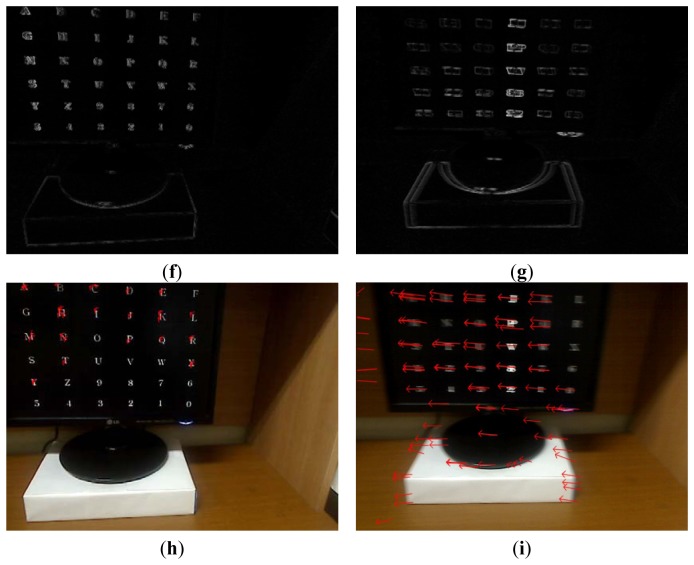
Example of a Type 2 error and the correct detection of head movement (person 9 listed in Table 6). (**a**) EEG signals. (**b**), (**d**), (**f**), and (**h**) respectively show signals obtained from FT of EEG signals, pixel difference, edge pixel difference, and motion vectors using LKT in case of Type 2 error. (**c**), (**e**), (**g**), and (**i**) respectively show signals obtained from FT of EEG signals, pixel difference, edge pixel difference, and motion vectors using LKT in case of correct detection of head movement.

Figure 8(a) shows an example of a Type 2 error and an example of the correct detection of a head movement (person 9 listed in Table 6). In the case of the user moving his/her head (Class 2), the FT-processed EEG signal has a lower amplitude at low frequency and a greater amplitude at high frequency. Although Figure 8(b) is actually the case of head movement (Class 2), the amplitude at low frequency is greater, which causes the final Type 2 error. On the contrary, Figure 8(c) is actually the case of head movement (Class 2), and the amplitude at low frequency is lower. In addition, the pixel difference and edge pixel difference values are greater and the number of motion vectors is large (Figure 8(e,g,i)), which causes the correct detection of head movement.

**Figure 9.**
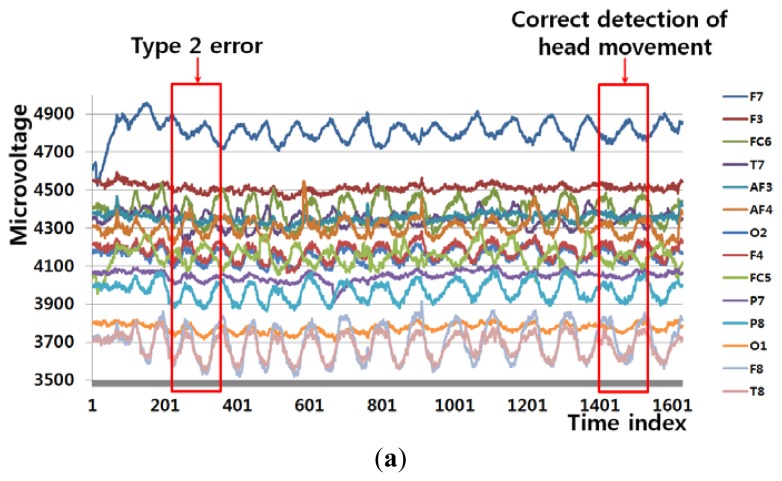

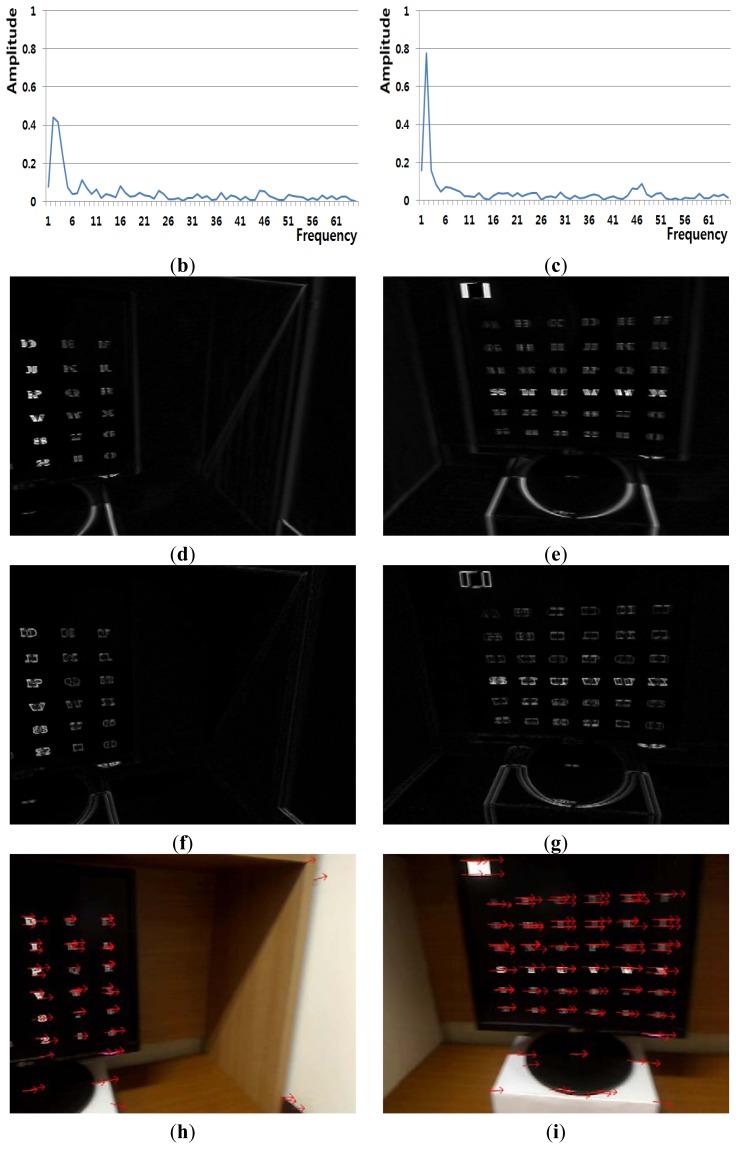
Example of a Type 2 error and correct detection of head movement (person 1 listed in Table 6). (**a**) EEG signals. (**b**), (**d**), (**f**), and (**h**) respectively show signals obtained from FT of EEG signals, pixel difference, edge pixel difference, and motion vectors using LKT in case of Type 2 error. (**c**), (**e**), (**g**), and (**i**) respectively show signals obtained from FT of EEG signals, pixel difference, edge pixel difference, and motion vectors using LKT in case of correct detection of head movement.

Figure 9(a) shows an example of a Type 2 error and an example of the correct detection of a head movement (person 1 listed in Table 6). Although it is the case of head movement, the amount of pixel, edge differences and motions of Figure 9(d,f,h) is smaller due to the severe rotation of user's head, which causes the Type 2 error. Although Figure 9(c) is actually the case of head movement (Class 2), the amplitude at low frequency is incorrectly higher. However, the pixel difference and edge pixel difference values are greater and the number of motion vectors is large (Figure 9(e,g,i)), which causes the correct detection of head movement. Therefore, the head movements are correctly detected by combining the frontal image features and the EEG features in the frequency domain using LDA and SVM.

We performed additional experiments to measure BCI performance in a 6 × 6 speller UI system, as shown in Figure 2(c). A total of five people participated in the experiments and each person performed five trials (without and with head movement).

**Figure 10.**
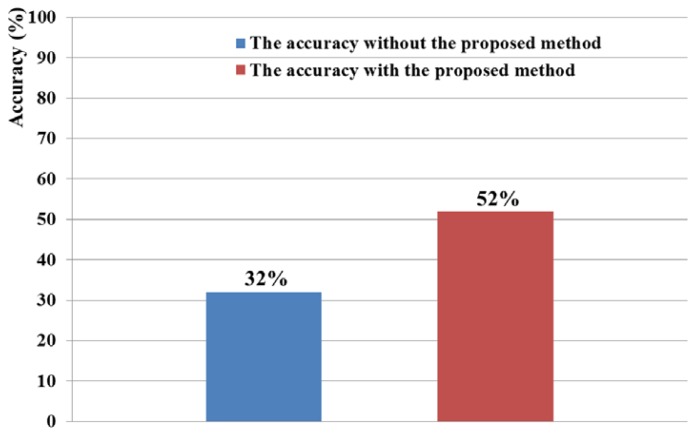
Comparisons of accuracies with/without the proposed method in speller UI system.

The 6 × 6 speller UI system has been widely used to measure BCI performance [20]. With a randomly given character (e.g., “R” at the upper-left corner in Figure 2(c)), each row or column is randomly highlighted 20 times. During that time, the maximum peak of the EEG signal is measured based on P300. The P300 is known as the measurement method of the positive component that happens between 200 and 500 ms after a specific stimulus [18,41]. Here, the highlighting of the row or column is regarded as the stimulus. If the maximum peak of EEG signal based on P300 exists in case that row or column including the same character to the given one ((e.g.,) “R” at the left-upper position of screen of Figure 2(c)) is highlighted, it is counted as the correct selection of character in speller UI system [18]. Based on this process, we measured the accuracy of selecting the correct character as the BCI performance. As shown in Figure 10, the accuracy of the P300-based BCI system with the proposed detection method of head movement is higher than that without our method. Rejecting the large sections of EEG data (which are determined as data including the noises by head movement) causes the reduction of the EEG data which can be used for further process. In order to solve this problem, we need to develop a method that regenerates the EEG data of the rejected sections, based on the data in the accepted sections.

## 4. Conclusions

We propose a new method that combines an EEG acquisition device and a frontal viewing camera to detect the EEG signals which include the noises caused by the user's head movements. We compared the accuracies in detecting the head movements based on the features of the EEG signals in the frequency and time domains and the motion features of the images captured by the frontal viewing camera. The features of the EEG signals in the frequency domain and the motion features captured by the frontal viewing camera were selected as the optimal ones. The dimension reduction of the features and feature selection are performed using LDA. The combined features are used as the inputs to the SVM, which enhances the accuracy in detecting the head movements. Our future work plans include the development of a scheme that combines additional information such as the user's gaze position, electrooculography (EOG), and electromyography (EMG) to enhance the accuracy in determining the head movement.

## Figures and Tables

**Table 1.** Comparison of previous and proposed methods to detect EEG artifacts caused by head movements.

**Category**		**Method**	**Advantages**	**Disadvantages**
Using single modality	Using only the EEG features [11–14]	EEG signals are analyzed to detect the head movements or eye blinks.	Acquiring EEG signals is faster than acquiring images with a frontal viewing camera.	The detection of the EEG artifacts is less accurate with a single modality than with multiple modalities.
	Using only the frontal image features [10]	Head movements are detected based on the image sequence captured by the frontal viewing camera.	Detecting head movements is more accurate when using this method than when using only the EEG features.	
Using multiple modalities	Using signals from both EEG and another sensor [15,16]	Signals from both the EEG and another sensor (gyroscope [15] or accelerometer [16]) are used to detect head movements.	Detecting head movements is more accurate with multiple modalities than with a single modality.	Methods using gyroscopes or accelerometers can distinguish fewer types of head motions than methods using frontal viewing cameras.
	Using both EEG and frontal image features (proposed method)	Both EEG signals and frontal image features are used to detect head movements.		Frontal viewing cameras can acquire images slower than EEGs, gyroscopes, or accelerometers can acquire signals.

**Table 2.** List of features.

Features from EEG signals	Frequency-domain features (12 features)	The normalized magnitudes in the frequency domain at the corresponding frequency bands (3–15 Hz, 4–6 Hz, 5–7 Hz, 6–8 Hz, 7–9 Hz, 8–10 Hz, 9–11 Hz, 10–12 Hz, 15–30 Hz, 20–30 Hz, 51–64 Hz, 59–61 Hz) obtained by using the Fourier transform
	Time-domain features (13 features)	Kurtosis, Skewness, RMS amplitude, zero-crossing, minimum, maximum, variance of first derivative, variance of second derivative, zero-crossing of first derivative, zero-crossing of second derivative, activity, mobility, complexity
Features from images captured by the frontal viewing camera	Pixel difference, edge pixel difference, average magnitude of the motion vectors, number of motion vectors (4 features)	

**Table 3.** Type 1, Type 2, and average errors in testing data using LDA.

**Combined features**	**Type 1** **error (%)**	**Type 2** **error (%)**	**Average** **error (%)**
EEG features in the frequency domain	35.49	28.41	31.95
EEG features in the time domain	23.28	23.34	23.31
EEG features in the frequency domain + EEG features in the time domain	21.87	26.67	24.27
Frontal image features + EEG features in the time domain	7.21	20.54	13.88
Frontal image features + EEG features in the frequency domain	1.73	4.96	**3.35**
Frontal image features + EEG features in the time domain + EEG features in the frequency domain	2.59	12.14	7.37

**Table 4.** Type 1, Type 2, and average errors in testing data using LDA and SVM.

**Combined features**	**Type 1** **error (%)**	**Type 2** **error (%)**	**Average** **error (%)**
Frontal image features + EEG features in the time domain	9.78	6.54	8.16
Frontal image features + EEG features in the frequency domain	4.55	1.89	**3.22**
Frontal image features + EEG features in the time domain + EEG features in the frequency domain	10.49	3.28	6.89

**Table 5.** Average error rate in testing data using only SVM.

**Combined features**	**Average error (%)**
Frontal image features + EEG features in the time domain	7.04
Frontal image features + EEG features in the frequency domain	3.49
Frontal image features + EEG features in the time domain + EEG features in the frequency domain	5.66

**Table 6.** Type 1, Type 2, and average errors for each subject when using both frontal image features and EEG features in frequency domain with LDA and SVM.

	**Type 1 error (%)**	**Type 2 error (%)**	**Average error (%)**
Person 1	3.17	1.6	2.39
Person 2	9.18	2.44	5.81
Person 3	1.64	1.14	1.39
Person 4	12.02	0.79	6.41
Person 5	0.54	5.64	3.09
Person 6	6.36	0.45	3.41
Person 7	4.57	0.07	2.32
Person 8	0.26	3.02	1.64
Person 9	3.6	3.3	3.45
Person 10	3.74	0.07	1.91
